# SG-APSIC1074: Trend of ‘ESKAPE’ and their susceptibility changes for meropenem and levofloxacin during the pandemic at Sardjito Hospital Yogyakarta Indonesia

**DOI:** 10.1017/ash.2023.7

**Published:** 2023-03-16

**Authors:** Andaru Dahesihdew, Yunan Nadhif Fanani

**Affiliations:** Sardjito Hospital, Indonesia; Yogyakarta, Sardjito Hospital, Indonesia

## Abstract

**Objectives:** The bacteria in the ‘ESKAPE’ group are monitored due to their ability to resist antibiotic action. During the COVID-19 pandemic at our hospital, the usage of meropenem and levofloxacin as the empirical treatment for bacterial pneumonia increased and might have contributed to the antimicrobial resistance problem. In this study, we evaluated the ESKAPE group infection rates and their susceptibility to antibiotics in Dr. Sardjito Hospital, a referral and academic hospital in Yogyakarta, Indonesia. **Methods:** Data for ESKAPE pathogens in 2019–2021 were taken from the microbiology laboratory of Dr. Sardjito Hospital and were evaluated. **Results:** The proportion of ESKAPE isolates among positive cultures during 2019–2021 slightly increased from 49.4% to 48.4% to 50.7% each year (*P* > .05). The dominant ESKAPE infections were pneumonia, bloodstream infection, and urinary tract infection by *K. pneumoniae*, and wound infection by *P. aeruginosa*. The susceptibility pattern of ESKAPE to meropenem decreased from 72% in 2019 to 68% in 2020 but increased to 84% in 2021. To levofloxacin, the susceptibility pattern was decreased in a fluctuating trend from 68% in 2019 to 33% in 2020 and to 39% in 2021. During the COVID-19 pandemic (2020–2021), the pattern of ESKAPE infections was similar to that of 2019. In descending order, the frequency rank was *K. pneumoniae, P. aeruginosa, A. baumannii, Enterobacter spp*, and *S. aureus*. The proportions of MDR isolates increased from the prepandemic period to the COVID-19 pandemic era for *E. faecium* (from 5% to 24.4%), for *A. baumannii* (from 9.6% to 38.5%), and for *P. aeruginosa* (from 7.4% to 13.5%) (*P* < .05). These patterns did not differ between non–COVID-19 patients and COVID-19 patients. These results highlight the general impact of overused antibiotics beyond COVID-19 patients. Usage of watched and restricted antibiotics must be more controlled because bacterial coinfection and superinfection in COVID-19 patients was relatively low. **Conclusions:** During the COVID-19 pandemic, ESKAPE infections increased and their susceptibility to meropenem and levofloxacin decreased. Tight control of antibiotic usage is needed.

